# DRIFTS Sensor: Soil Carbon Validation at Large Scale (Pantelleria, Italy)

**DOI:** 10.3390/s130505603

**Published:** 2013-04-26

**Authors:** Filippo Saiano, Giacomo Oddo, Riccardo Scalenghe, Tommaso La Mantia, Franco Ajmone-Marsan

**Affiliations:** 1 Dipartimento Scienze Agrarie e Forestali (SAF), Università degli Studi di Palermo, viale delle Scienze ed. 4, Palermo I-90128, Italy; E-Mails: giacomo.oddo@yahoo.it (G.O.); riccardo.scalenghe@unipa.it (R.S.); tommaso.lamantia@unipa.it (T.L.M.); 2 Dipartimento Scienze Agrarie, Forestali e Alimentari, Università degli Studi di Torino, Via Leonardo da Vinci 44-Grugliasco, Torino I-10095, Italy; E-Mail: franco.ajmonemarsan@unito.it

**Keywords:** carbon, nitrogen, DRIFT, PLS, Technosols, Mediterranean, anthropogenic soils

## Abstract

A fast and accurate measurement of soil carbon is needed in current scientific issues. Today there are many sensors suitable for these purposes, but choosing the appropriate sensor depends on the spatial scale at which the studies are conducted. There are few detailed studies that validate these types of measures allowing their immediate use. Here it is validated the quick use of a sensor in execution at Pantelleria, chosen for size, use and variability of the parameter measured, to give an operational tool for carbon stocks studies. The DRIFT sensor used here has been validated in the first 60 cm of the soil of the whole island, and it has shown predictivity higher than 90%.

## 1. Introduction

Reflectance spectroscopy (RS) has successfully used for the discrimination of soil types from satellite, and multi spectral and aircraft hyperspectral data [1–3]. Similarly, it was used for quantitative soil–landscape modeling [4,5], precision agriculture [6,7], tracing sediments [8] and for soil carbon (SC) monitoring [9].

The ability of RS to provide non-destructive rapid prediction for soil physical, chemical and biological properties in the laboratory has been demonstrated [4]. Despite these indications of the potential of the technique, there are relatively few examples of the application of RS for non-destructive assessment of soils [10,11], soil and crop physical and biochemical properties. Although the near-infrared (NIR) range (800–2,500 nm) is still the most widely used, mid-infrared spectroscopy (MIRS) is becoming increasingly common due to the specificity toward the functional groups of the absorbance bands in that spectral range. Fourier transformed infrared (FTIR) spectroscopy, and in particular diffuse reflectance FTIR spectrometry (DRIFTS) analysis, has been used extensively in studies on organic molecules in soils and a quite large variety of bands characteristic of molecular structures/functional groups have been identified [11,12]. Moreover, the DRIFTS analysis of undiluted soil samples can also be used to assess soil organic matter (SOM) composition also in small sample sets, if mirror reflection and (mineral) interferences are considered [4], and in natural environments, sample preparation for DRIFTS is much simpler than for transmission IR spectroscopy and interferences due to water adsorption are reduced and resolution is improved [12,13]. DRIFTS, along with partial least squares (PLS) algorithms, provides statistical models to quantify soil properties [14]. The statistical treatment of the principal component regression (PCR) and partial least squares regression (PLSR), in fact, are the most common techniques used for spectral calibration and prediction [15]. The methodology and accuracy assessment of current remote sensing analyses can explore land cover at 30 m spatial resolution using satellite data as reference material. Some soil attributes has been validated using mid-infrared diffuse reflectance spectroscopy sensors at small scale [16] and a large scale [17].

Diverse entities are working on future markets for trading of greenhouse gases (GHG) emissions allowances (e.g., the Carbon Finance Unit at the World Bank), but still today one of the impediments in growing up of these voluntary markets is the cost of information needed to quantify benefits on a site-specific basis. A method for rapid assessment of the carbon content in the soil also takes great interest for the purposes of a possible inclusion, for instance, by the European Union among the activities eligible in the second commitment period of the Kyoto Protocol (Art 3.4) [18].

Here it is applied DRIFTS-PLS to quantify SC contents comparing the elemental determination for bulk soils and soil fractions in a small and homogeneous area, Pantelleria Island, with a carbon-free substrate to investigate the changes in SC stock as a function of changes in land cover.

## 2. Materials and Methods

Pantelleria (36°N 12°E), the major satellite island of Sicily (83 km^2^), is the summit of a partially revealed volcano located 100 km S of Sicily and 70 km N of Tunisia on the axis of the Sicily Channel Rift Zone. The volcanic edifice is a stratovolcano emerging above sea level at 836 m, which is covered by tephra layers including basalts, felsic lavas, and trachytes and pantellerites tuffs erupted mainly 300 ka and 50 Ka producing ignimbritic deposits up to 20 m thick [19]. The island is semi-arid with a typical Mediterranean climate (MAT 18 °C, MAP 409 mm) with monthly average temperatures fluctuating between 12 and 26 °C [20]. First archaeological facts date 35 Ka ago although the island remained almost uninhabited until the Carthaginians occupied it, followed by the Romans and later by the Arabs. Pantelleria, in fact, provided obvious stop-over sites on the traditional North-South axis of the wheat and non-food good trade [20–22]. Pantelleria is characterized by a uniqueness of its landscape, in which natural elements do not prevail over the artifacts created by man, mainly its drywalls and cylindrical gardens. Pantelleria has been nearly totally terraced during centuries and cultivated with grapevine, caper, and olive trees. Adaptive field fragmentation allow numerous small property owners to adapt to changing climate and cost of labor [23]. About 80% of these terraces are nowadays abandoned [18] and small areas of pristine forests and Maquis are rather widespread. Following the abandonment of agricultural activity, which took place from the middle of the last century, areas of considerable extent have been affected by succession processes [24].

Mineralogy investigations [25] have shown that the major components of all soils from Pantelleria are directly inherited from the volcanic carbon-free bedrocks. Due to the presence of a certain amount of artefacts, or technic hard rock the classification of our soils is rather homogeneous, most of them (69 out of 78) belonging to Vitric Technosols (skeletic) [26], while prefix qualifiers in few (6/78) soils are ekranic or umbric. For purpose of comparison, few samples (3/78) from Umbric Leptosols and Umbric Andosols were considered in our study. The undisturbed soil used in this study were collected from Ap horizons, 100 cm^3^ of samples were collected with a metal cylinder at depth 10, 20, 30, 40 and 60 cm. The volume and the weight of the skeleton of each sample were measured simultaneously by immersion in a known volume of distilled water in a graduated cylinder [27]. The fine-earth samples were weighed and then dried at a temperature of 105 °C until reaching a constant weight. Later they were crushed and sieved at 2 mm, then stored at −18 °C.

Electrical conductivity and pH measurements were carried out with a multiparametric probe (Hanna Instruments HI 991300, Woonsocket, Rhode Island). The pH was measured in three distinct suspensions of 1:2.5 soil: solution, water and 1M KCl, and 1:50 1M NaF. The measurements of total amount of C and N on the solid phase were performed on 0.5 mm grounded samples using an elemental analyzer (Fisons EA1108, Ipswich, United Kingdom). The range extension in content of total C and N for these samples provided a good test on the ability of MIRS to quantify soil C and N.

Infrared radiation reflected from the surface of the soil samples was measured by a DRIFT module on a FT-IR spectrometer ALPHA (Bruker Optics, Milano, Italy). The analysis was conducted (i) on the soil (<2 mm) crushed in an agate mortar and (ii) on 0.1 g of the same soil dried at 40 °C and crushed with the same amount of KBr (Fluka FTIR grade). For each of the two procedures duplicate measurements were run. All measured carbon concentrations were then corrected to an oven-dry equivalent mass. DRIFTS–PLS predicted the C and N contents more precisely (larger *R^2^*, smaller error of prediction) for KBr-diluted samples than for neat samples, therefore, it is used KBr-diluted samples. The spectra were acquired by 24 scans performed in the range between 7,000 cm^−1^ and 375 cm^−1^ at a resolution of 4 cm^−1^ (air was used as a background). The acquisition and processing spectral as well as the subsequent statistical treatments and the multivariate calibration method used for quantitative spectral analysis were performed with the Bruker software OPUS, statistical package OPUS QUANT2 6.5. The PLS method reduces a set of spectroscopic calibration data into a set of loadings, taking into account the spectral variance and the predetermined value of each of the samples in the calibration data set. To predict the value of a new sample, its spectrum is fitted with the same loadings as the calibration set, and the properties are predicted from the regression coefficients of the PLS model. The IR spectra of samples were elaborated with OPUS QUANT2 software to determine the calibration function by a multivariate calibration by which the software extrapolates the chemiometric model which is successively cross validated. The quality of results was evaluated by *R^2^*, *bias* and residual prediction deviation (*RPD)* parameters. The coefficient of determination (*R^2^*) gives the percentage of variance present in the true component values, which is reproduced in the prediction. *R^2^* approaches 100% as the predicted concentration values approach the true values. The *bias* is a systematic deviation of the measured (predicted) values from the true value due to a particular measurement method. In the research, it is the difference between the average true value and the average measured value of the validation set samples. The RPD is the ratio of standard deviation to standard error of prediction which indicates the standard deviation of all bias-corrected measured values from the true value. The most capable QUANT2 method is the one with the highest RPD value and a bias value as close as possible to zero.

## 3. Results and Discussion

**Figure 1.**
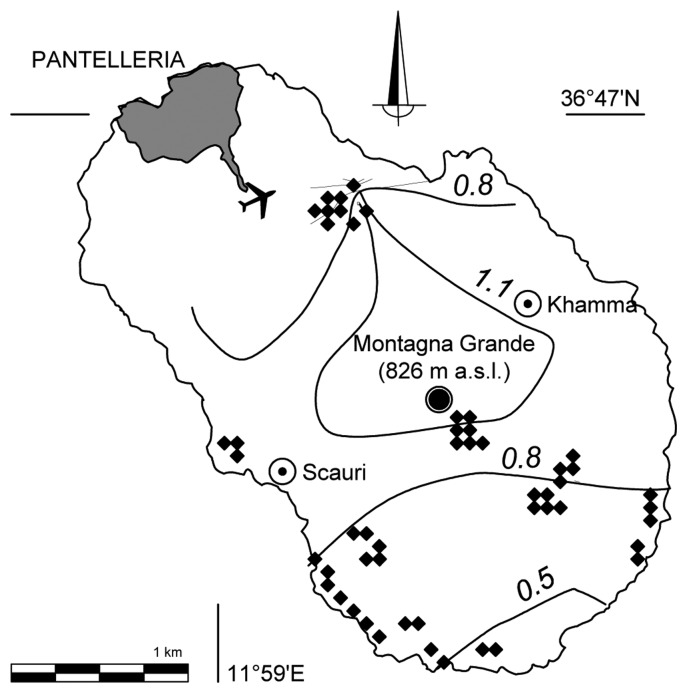
Sites where soils were sampled for this study, where lines indicate the percent of soil total carbon at depth of 30 cm. The grid is irregular as sampling was carried out for extracting the highest C variability, regionalized by the use of soils. In particular, northern sampling points collected the greater variability of soils in terms of crops. In the South, the variability is given by the natural environment of the Mediterranean vegetation, while towards West by different gradients of human pressure. A regular sampling grid would not have correctly interpreted this intrinsic variability.

Figure 1 reports the distribution of the sampling points on Pantelleria map while Table 1 describes the input data for the full soil set showing that while the ranges of nitrogen, carbon and electrical conductivity vary widely, bulk density and pH values were within relatively narrow ranges.

The distributions of the data for C, N and EC are right-tailed and leptokurtic, being asymmetric toward small values and more acute peak near its mean compared with the standard normal distribution. The soil variability is mainly due to anthropogenic changes. The cultivation on terraces and the subsequent abandonment are the reason for the high variability in the carbon content of the soil even at a detailed scale.

**Figure 2.**
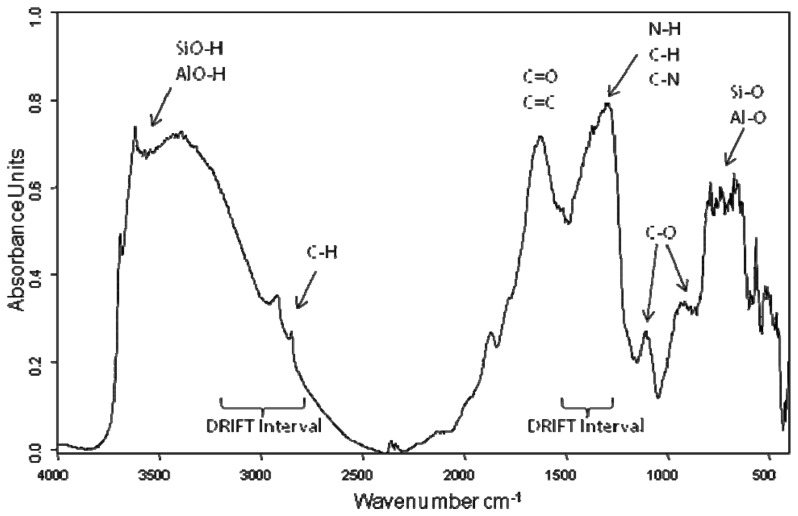
DRIFT spectrum with assignment of principal bands investigated and the spectral width regions used for the PLS analysis of the selected soils.

Mid-infrared spectra contain information on major functional moieties of organic matter, such as alkyl C, C-O-C from polysaccharides, aromatic carbonyl bands, aromatic C=C stretching vibrations, and C=O from carboxylic acids, aldehydes and ketones and so on. In the range 3,700–3,500 cm^−1^ were assigned the sharp peaks of SiO-H and AlO-H stretching. The broad adsorption band in the range 3,500–2,500 cm^−1^ was indicative of the presence of the O–H and N–H groups, widely involved in hydrogen bonds. It was possible to see overlapped also the weak peaks around 2,900 cm^−1^ assigned to C–H stretching of CH_3_ and CH_2_ groups. The band at 1,730 cm^−1^ was generally due to C=O stretching of COOH and other carbonyl groups [10–13]. The bands at around 1,650–1,600 cm^−1^ were attributed to several group vibrations including aromatic C=C and C=O stretching of amide I groups (in protein like compounds) and the absorption at about 1,540 cm^−1^ was ascribed to N-H deformation and C-N stretching of amides (amide II group) [10–13]. The shoulder appearing at 1,460–1,450 cm^−1^ was by aliphatic C-H deformations and/or aromatic ring vibrations. The broad band in the range at 1,450–1,220 cm^−1^ was attributed to O-H deformation, C-O stretching of phenolic OH and C-H deformation of -CH_2_ and -CH_3_ groups together C-O stretching and O-H deformation of carboxyls and C-O stretching of aryl ethers, and finally a band between 1,150 and 1,000 cm^−1^ was due to C-O stretching of polysaccharide or polysaccharide-like substances [12,13]. No band in the range 2,650–2,500 cm^−1^, attributable to carbonate group was detected in our spectra in agree with chemical determination. Figure 2 reports a typical spectrum obtained in DRIFTS measurements while in Table 2 were summarized the principal structural assignments.

However, in DRIFT spectra of bulk soils and/or soil fractions, many organic signals are superimposed on signals from minerals (e.g., Si-O bonds from silicates at around 1,030 cm^−1^ interfere with the signal of polysaccharides) and water. This makes direct peak integration almost impossible, with the exception of the alkyl band, which has already been used sometimes for direct quantification of alkyl C. For these reasons, the chemical information is typically not directly accessible but has to be deciphered by multivariate statistical analysis. The spectral information contained in the loading weights is thereafter directly related to the property of interest. Thus, PLSR not only delivers quantitative information, but the spectral information used for the predictions can also be interpreted chemically and checked for plausibility. Table 3 presents the full cross-validation results for C and N.

For the optimized PLS calibrations, some spectral data points, not contributing to the relationship between spectra and the specific soil property, were removed from the final calibration models. Such data filtering resulted in some slight improvement of the prediction accuracies. The *R*^2^ values were 0.951 for C and 0.945 for N. These values agree reasonably well with those reported by other researchers in the USA, Australia [28,29] and particularly with other three Italian islands [17]. The RPD values (SD/RMSECV, where SD is standard deviation and RMSESCV is root mean square error of cross-validation) for C and N (4.49 and 4.28, respectively) were consistent with a good analytical accuracy. A significant degradation of infrared PLS accuracy and precision for C by carbonate interference was reported by Reeves [30], but this effect was obviously not observed with our data.

**Figure 3.**
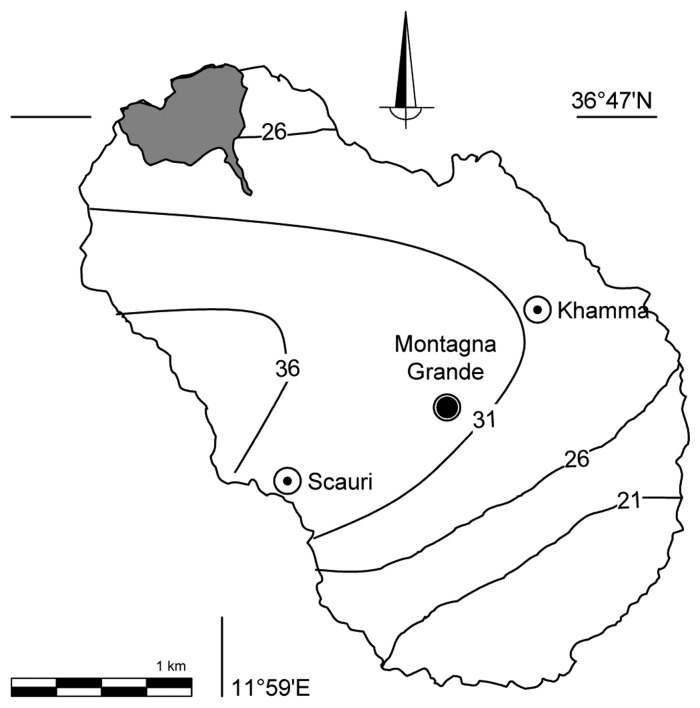
Lines indicate the error of predicted soil total carbon at any depth within 60 cm. Kriged C content is given as a maximum percentage of the error in the extrapolation of the predicted values (n = 89, standardised experimental variogram max lag 3,900 m, direction −16, tolerance 90, step amount 30, lag width 130, nugget 0.046, covariance 0.06, skewness 1.3).

In accordance with the basic principles of sample selection for calibration, as suggested by Naes [31], the results obtained indicated that the strategy of samples selection, *i.e.*, the large variability of the chemical parameters studied and of the sampling depth, as well as of the spectra in the model and the resulting coverage of the PLS score space within the complete sample set, ensuring that all samples from the island were adequately represented, was effective. This suggests that proper selection of samples representative of this environment (Figure 1) is more important than solely the number of samples used in the calibration [17,31]. Moreover, the DRIFTS frequency regions in which the program gets the best correlation coefficients (Table 3), were consistent with those expected to be more significant for C and N. It is calculated the error in the measurement of carbon and nitrogen obtained from the estimates with DRIFTS. In the case of nitrogen the estimation error is rather homogeneous spatially and always less than 30%. In the case of carbon (Figure 3) with the exception of the volcanic edifice in the terraces closer to the coast the error is around 25%. Soil carbon of the western terraces, from the Western coast by Scauri, can be estimated with an error of less than 40%.

**Figure 4.**
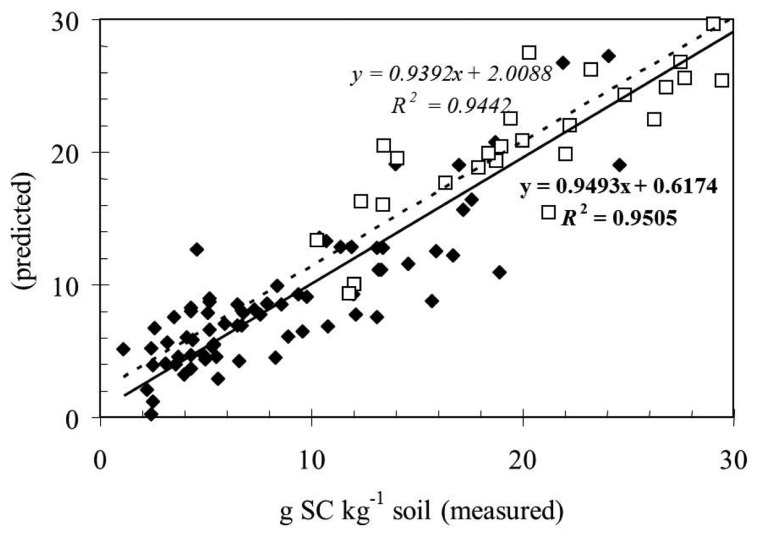
Actual values and predicted values (g kg^−1^) comparing our data for Pantelleria (filled diamonds) and the data obtained by D’Acqui *et al.* [17] in the island of Pianosa (open squares) in the range of three percent carbon content of soils.

However, the implications of these results, using the overall samples, were more robust models for C and N amounts determinations with a good accuracy. This would suggest that prediction models for these analytes built from the individual island may possibly be successfully used to predict larger soil sets. This possibility was tested comparing the DRIFT data of another geologically different Mediterranean island [17]. The results were illustrated in Figure 4 where the agreement between the data is very encouraging.

## 4. Conclusions

Detection of soil carbon evolution has a particular value for policy intervention and for carbon based emissions trading [32], so *in situ*, time-efficient and cost-effective, C measurement methods will be developed to facilitate the prediction on stocks through the on-going generation of spectral libraries from diverse soils and climatic conditions for the intention of validation [33]. The traditional methodologies used for measuring soil carbon content and other soil properties are time-consuming, analytically expensive, mainly sample-destructive and costly. Thus developing of new sensors for quick and less expensive are important. Several researches have demonstrated that DRIFTS is accurate and produces more robust calibrations than other technique [4,7,8,16,17,29]. DRIFTS have tremendous potential for the rapid and inexpensive determination of soil carbon, and other soil parameters. The DRIFTS PLS method of soil property predictions was shown to be suitable for the characterization of typical soils of islands in a Mediterranean environment [17]. It is applied DRIFTS-PLS to quantify soil C and N contents compared to elemental determination for several bulk soils. Our aim, to evaluate if a local calibration with satisfying prediction quality is attainable, in particular for deciphering and drawing the C or N spatial patterns at the field scale, was obtained. Calibration models for C and N attained *R^2^* values of 0.951 and 0.945 respectively. Also these results, achieved by DRIFTS and PLS regression, confirmed that infrared methods are capable of discriminating between the variation in chemistry and composition in soils with a potential advantage of speed and low cost. Obviously, every PLS calibration model may be further expanded with additional soils and data to allow more accurate and detailed characterization of soil variability.

Total carbon stock in the first 30 cm of soil of Pantelleria is about 230,000 Mg, and the error of the estimates using the calibrated DRIFT sensor directly spans from 6 to 10 Mg SC ha^−1^. Ten years ago, Antle *et al.* [34] esteemed costs of errors in standard sampling for carbon accounting: those costs ranged from 0.01 to a maximum of 8 EUR per tonne, based on the sample size required to achieve a minimum 10% sampling error (obviously, the costs increase as the sampling error approaches zero). Extrapolating these hypotheses in Pantelleria, an annual accounting error costs would span from 2,000 to one million EUR in the zero error sampling scenario. The use of a quick and rather accurate sensor could reduce the total cost of carbon accounting by augmenting the intensity of sampling in time and space.

## Figures and Tables

**Table 1.** Reference laboratory data statistics for the samples used in this study (N = 89).

**Parameter**	**N_tot_**	**C_tot_**	**Bulk Density**		**pH**		**Electrical Conductivity**

	**(%)**	**(%)**	**(g cm^−3^)**	**(KCl)**	**(H_2_O)**	**(NaF)**	**(dS m^−1^)**
Range	0.39	8.3	0.9	3.5	2.5	2.7	0.4
Mean	0.09	1.4	†1.1	5.7	6.9	8.2	0.1
Standard deviation	0.08	1.5	0.2	0.7	0.5	0.5	0.1
Skewness	2	3	0	0	−1	2	2
Kurtosis	4	8	0	1	1	3	3

† First five cm of soil 1.0 ± 0.3, 10 cm 1.1 ± 0.2, 20 cm 1.1 ± 0.2, 30 cm 1.1 ± 0.2, >40 cm 1.0 ± 0.2.

**Table 2.** Structural assignments of the main signals featured in a typical spectrum (Figure 2).

**Wavenumber cm^−1^**	**Structural Assignment**
3,700–3,600	OH of clay and Fe oxides
3,400–3,100	O–H and N–H
2,980–2,850	aliphatic C–H
1,730–1,620	C=O carbonylic
1,620–1,600	C=C
1,565–1,480	carboxylate COO^−^
1,470–1,300	C–H and C=C
1,180–1,000	hydroxylic C–OH, Si-O Al–O
800–600	Si–O Al–O

**Table 3.** DRIFT settings.

**Parameter**	**Wavenumbers cm^−1^**	**Functional Group**	**Slope**	**Bias**	***R*^2^**	**RPD**
Carbon	3,160–2,810	C–H	0.949	0.042	0.951	4.49
	1,510–1,300	C–H				
Nitrogen	3,350–2,620	N–H	0.935	0.004	0.945	4.28
	1,485–1,300	C–N				

R^2^ = coefficient of determination; RPD = residual predictive deviation.
